# Radiomics-Based Machine Learning for Sarcopenia Detection in Abdominal and Low-Dose CT

**DOI:** 10.3390/diagnostics16111617

**Published:** 2026-05-25

**Authors:** Soo-Been Kim, Young Jae Kim, Kwang Gi Kim

**Affiliations:** 1 Medical Devices R&D Center, Gachon University Gil Medical Center, Incheon 21565, Republic of Korea; rlatnqls22ee@gmail.com; 2 Gachon Biomedical & Convergence Institute, Gachon University Gil Medical Center, Incheon 21565, Republic of Korea; kimyj10528@gmail.com; 3 Department of Biomedical Engineering, College of Medicine, Gachon University Gil Medical Center, Incheon 21565, Republic of Korea

**Keywords:** sarcopenia, radiomics, low-dose CT, abdominal CT, machine learning

## Abstract

**Background**: Sarcopenia, characterized by progressive loss of skeletal muscle mass and function, is becoming increasingly prevalent with the global population aging. Computed tomography (CT) is widely used for muscle assessment; however, concerns regarding radiation exposure have prompted interest in lower-dose imaging protocols. This study investigated the performance of radiomics-based machine learning (ML) models for sarcopenia detection using abdominal CT (APCT) and low-dose CT (LDCT). **Methods**: Radiomics features were extracted from CT images following skeletal muscle segmentation, and ML models were developed using logistic regression, support vector machine, and random forest. Model performance was evaluated using fivefold cross-validation with out-of-fold predictions. **Results**: The random forest model demonstrated the best performance among the evaluated models, achieving an area under the receiver operating characteristic curve of 0.720 (95% CI: 0.532–0.881) for APCT and 0.692 (95% CI: 0.573–0.801) for LDCT. Model interpretation using SHapley Additive exPlanations analysis identified several intensity-based radiomics features, including TotalEnergy, as important contributors to sarcopenia prediction. **Conclusions**: These findings suggest that radiomics features derived from LDCT images may provide useful information for sarcopenia detection. Because LDCT is widely used in clinical settings such as lung cancer screening, radiomics analysis of LDCT images may offer an additional opportunity for opportunistic sarcopenia assessment.

## 1. Introduction

Sarcopenia is a progressive systemic disorder associated with decreased skeletal muscle mass, strength, and physical function and increased risk of mortality [1,2,3]. The global population is aging rapidly, and the number of individuals aged 65 years or older is projected to reach approximately 1.5 billion by 2050 [4,5]. Sarcopenia prevalence is rising, with projected cases increasing from ~50 million to 200 million [6]. Given its increasing health impact, sarcopenia is now recognized as a major public health concern requiring active investigation and management [7].

Recently, various machine learning (ML)-based approaches have been applied to sarcopenia diagnosis using different types of data sources, including medical imaging and physiological signals [8,9]. In this regard, radiomics is a computational technique that extracts quantitative features, such as texture and morphological attributes, from medical images to characterize diseases. It has been widely used in the diagnosis and prognostication of conditions such as cancer and sarcopenia [10,11,12]. Furthermore, radiomics, in combination with ML techniques, has been applied for automated assessment of muscle quantity and improved diagnostic modeling of various diseases [13,14,15]. Accurate quantification of muscle mass is essential for diagnosing sarcopenia, and imaging modalities such as dual-energy X-ray absorptiometry (DEXA), computed tomography (CT), and magnetic resonance imaging (MRI) are commonly used [16].

Blanc–Durand et al. developed a deep learning (DL) ensemble model based on a U-Net architecture trained on 1025 CT slices for muscle segmentation and achieved a Dice Similarity Coefficient (DSC) of 0.97 on an independent test set of 500 images [13]. Wang et al. analyzed radiomic features from PET/CT images of 178 patients and evaluated them using an ML algorithm (support vector machine), achieving 79.2% sensitivity, 83.3% specificity, and 78.3% accuracy [14]. Onishi et al. trained a sarcopenia detection AI using the EfficientNetV2-XL model on 3096 CT images and reported a sensitivity of 82.3% and specificity of 98.1% [17]. Dong et al. developed a LightGBM model trained on CT images from 99 patients and optimized it using Bayesian techniques, achieving a sensitivity of 83.3%, specificity of 94.4%, accuracy of 90.0%, and an area under the receiver operating characteristic curve (AUC) of 0.889 [18]. Yin et al. trained a Gradient Boosting Classifier (GBC) using a dataset of 11,661 middle-aged and older adults and achieved an AUC of 0.831, an accuracy of 88.9%, a recall of 44.1%, and an F1-score of 0.458 [19]. Ryu et al. employed DL models to analyze chest X-ray images from 926 patients and achieved an AUC of 0.813 [20]. Zhang et al. developed a Wide and Deep (W&D) model using 4057 training samples from the West China Health and Aging Trend (WCHAT) cohort and 553 external validation samples from the Xiamen Aging Trend (XMAT) study, achieving an AUC of 0.970 and accuracy of 0.911 [21].

Although CT imaging remains the dominant modality for muscle quantification in sarcopenia research, emerging studies have explored alternative imaging approaches, such as PET/CT or X-ray, for muscle quantification and sarcopenia diagnosis. Although CT provides rich anatomical information, concerns regarding radiation exposure remain, particularly in vulnerable populations [22,23]. To address this issue, low-dose CT (LDCT) has been proposed as a viable alternative that offers reduced radiation exposure while maintaining its clinical value [24]. LDCT is widely used for lung cancer screening and offers clinically relevant health information beyond its primary use [25]. However, LDCT suffers from high noise levels and reduced spatial resolution [26]. To address these limitations, several dose optimization and image reconstruction strategies have been proposed. Traditional approaches mainly focus on reducing X-ray exposure by lowering tube current and shortening acquisition time [27]. In addition, advanced reconstruction techniques based on compressed sensing and reduced projection sampling have been developed to further improve image quality in LDCT [28]. Recent studies have explored radiomics-based approaches to mitigate these limitations and enhance diagnostic performance using LDCT data [29,30,31].

Despite the growing interest in radiomics for sarcopenia assessment, most studies have primarily focused on standard-dose abdominal CT (APCT). Relatively few studies have investigated radiomics-based analysis using low-dose CT (LDCT) images for sarcopenia detection.

Therefore, this study aimed to develop and evaluate ML models based on radiomic features extracted from APCT and LDCT images. Model performance was assessed using metrics including AUC, accuracy, sensitivity, specificity, and F1-score. In addition, model interpretability was explored using SHapley Additive exPlanations (SHAP) analysis to identify important radiomic features associated with sarcopenia prediction [32].

By examining radiomics-based ML models derived from both APCT and LDCT images, this study sought to investigate whether LDCT radiomics features may provide useful information for sarcopenia detection. Because LDCT is widely used in clinical settings such as lung cancer screening, radiomics analysis of LDCT images may offer an additional opportunity for opportunistic sarcopenia assessment.

## 2. Methods

### 2.1. Data Collection and Labeling

This study was conducted in accordance with the ethical principles of the Declaration of Helsinki and was approved by the Institutional Review Board (IRB) of Gachon University Gil Medical Center (IRB No. GAIRB2019-003, approval date: 4 January 2019). As this was a retrospective observational study, the requirement for informed consent was waived by the IRB.

CT data were retrospectively collected from patients who underwent abdominal CT (APCT) or low-dose chest CT (LDCT) at Gachon University Gil Medical Center. All imaging data were retrieved in Digital Imaging and Communications in Medicine (DICOM) format.

A total of 214 patients were included in this study, consisting of 75 patients in the APCT cohort and 139 patients in the LDCT cohort between 1 January 2015 and 30 November 2018. Detailed clinical characteristics, including age and sex distributions stratified by imaging modality and sarcopenia status, are summarized in Table 1.

CT scans were performed using single-energy CT, and dual-energy CT was not used in this study.

The APCT and LDCT cohorts were treated as independent populations stratified by imaging modality, with no overlap between groups. Radiomics feature extraction and subsequent model development were conducted separately for each cohort without dataset integration.

All DICOM images were used for radiomics analysis. To prevent data leakage [33] and ensure patient-level independence, a single representative CT slice per patient was selected for analysis. This 2D approach was adopted based on prior evidence showing a strong correlation between L3/L1 skeletal muscle cross-sectional area and whole-body muscle mass [34]. In addition, previous studies have demonstrated that two-dimensional radiomics can achieve performance comparable to three-dimensional volumetric analysis in CT-based muscle assessment [35]. Given that 2D representations can provide sufficient information for muscle assessment, this approach was employed in the present study for computational efficiency.

**Figure 1:**
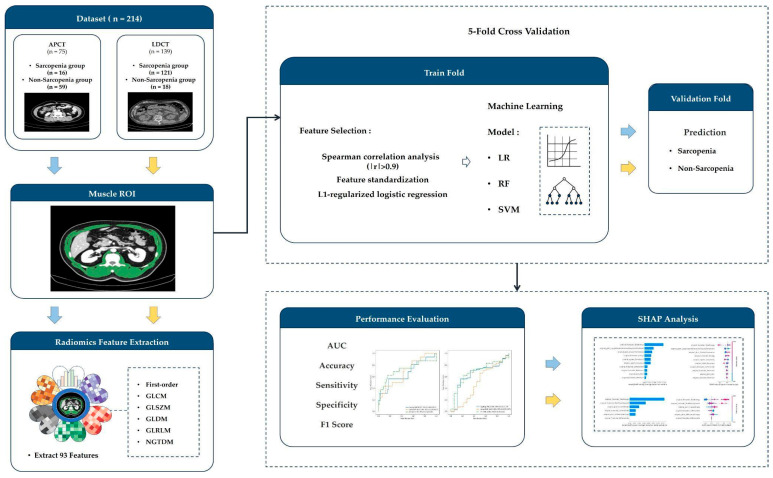
Flowchart of sarcopenia data collection and analysis: Data collection, data preprocessing, feature extraction, model training and validation, and model evaluation.

The overall workflow is illustrated in Figure 1.

Two board-certified radiologists, each with 10 years of experience in musculoskeletal radiology, independently annotated the regions of interest (ROIs) corresponding to the muscle areas on APCT and LDCT images. In cases of disagreement, consensus was reached through discussion.

The skeletal muscle area (SMA, cm^2^) was quantified using a custom-written Python script (version 3.8.2). SMA was calculated by summing the number of pixels within the manually annotated regions of interest (ROIs) and multiplying by the pixel surface area derived from image spacing.

The skeletal muscle index (SMI, cm^2^/m^2^) was subsequently calculated by normalizing SMA by the square of the patient’s height (m^2^).

Sarcopenia was defined based on the L3 muscle index (L3MI, cm^2^/m^2^), which was calculated by normalizing the cross-sectional area of the total skeletal muscle at the third lumbar vertebra (L3) by height, according to the international consensus criteria for cancer cachexia. Specifically, sarcopenia was defined as L3MI < 50 cm^2^/m^2^ in men and <39 cm^2^/m^2^ in women [36,37].

**Figure 2:**
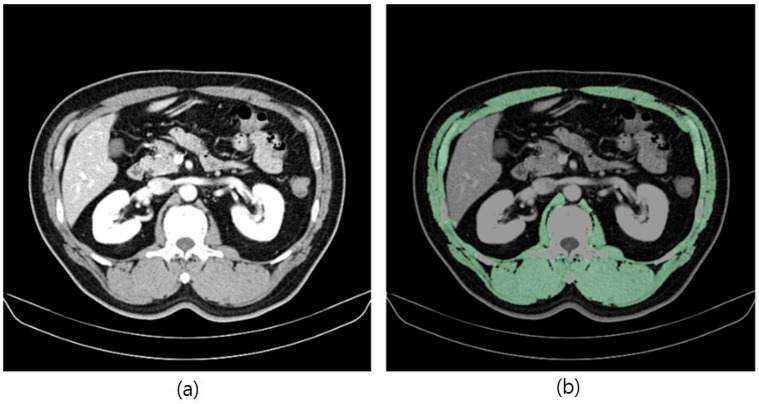
(**a**) APCT image and (**b**) APCT image with the overlaid muscle ROI.

The cross-sectional area at the first lumbar vertebra (L1) level, termed the L1 muscle index (L1MI), is strongly correlated with L3MI [38,39]. Accordingly, APCT images were used to diagnose sarcopenia at the L3 level, whereas LDCT images were assessed at the L1 level. Figure 2 shows an example of muscle segmentation in CT images: (a) the original APCT image and (b) the corresponding image with the segmented muscle ROI.

### 2.2. Training Environment

The experiments were conducted on a workstation equipped with an NVIDIA GeForce GTX 1660 GPU (NVIDIA, Santa Clara, CA, USA), Intel Core i7-10700 CPU (Intel Corporation, Santa Clara, CA, USA), and 32 GB of RAM. The ML workflows were implemented using the Python programming environment (version 3.8.2) on a Windows 10 Pro operating system.

### 2.3. Image Preprocessing

The image intensities were converted into Hounsfield Units (HU) by applying the Modality Lookup Table (Modality LUT) as defined in the DICOM standard [40]. Specifically, a rescaling intercept of −1024 and a rescaling slope of 1 were consistently applied to both APCT and LDCT images to ensure uniform HU conversion. Subsequently, the window center and width were adjusted using the Value of Interest (VOI) LUT transformation to optimize brightness and contrast for analysis.

**Figure 3:**
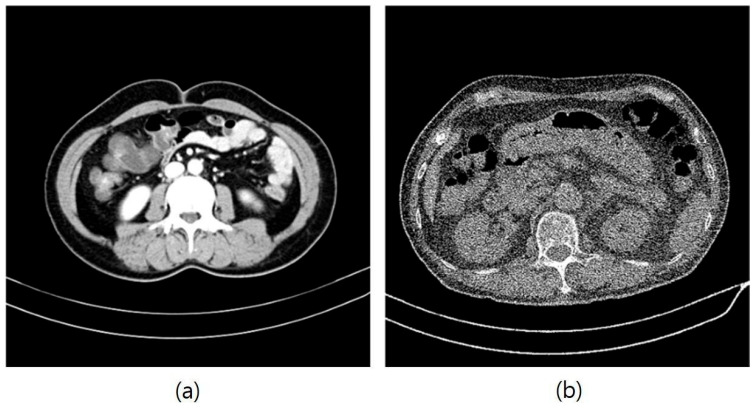
Examples of two types of images: (**a**) APCT image after HU conversion; (**b**) LDCT image after HU conversion.

The typical HU range for skeletal muscle tissue is approximately −29 to +150 HU [41]. Based on this range, window settings were configured to emphasize the muscle regions while preserving contrast with the surrounding tissues and avoiding excessive enhancement of bone structures. A uniform window center of 10 HU and a window width of 300 HU were applied to the APCT and LDCT images. Figure 3 shows examples of the APCT and LDCT images after transformation under the described windowing settings.

### 2.4. Radiomic Feature Extraction

Radiomics features refer to quantitative descriptors of tissue texture, shape, and density extracted from medical images that capture information often imperceptible to the human eye. These features are widely used to analyze the radiological characteristics of diseases [15].

In this study, regions of interest (ROIs) corresponding to muscle areas were manually delineated on CT images and used as masks for feature extraction. Radiomic features were extracted using the Python package PyRadiomics (version 3.1.0) [42]. Multiple categories of radiomic features were calculated, including first-order statistics, gray-level co-occurrence matrix (GLCM) features, gray-level run length matrix (GLRLM) features, gray-level size zone matrix (GLSZM) features, gray-level dependence matrix (GLDM) features, and neighboring gray-tone difference matrix (NGTDM) features [43].

Specifically, 18 first-order features, 24 GLCM features, 16 GLRLM features, 16 GLSZM features, 14 GLDM features, and 5 NGTDM features were extracted from APCT and LDCT images, resulting in a total of 93 radiomic features.

Feature extraction was performed using a bin width of 25. Intensity normalization was not applied. Radiomic features were calculated in two-dimensional mode (force2D = True, force2D dimension = 0). Image resampling was not performed during feature extraction. The default settings of PyRadiomics were used for all other parameters.

### 2.5. Feature Selection and ML Models

To reduce redundancy among radiomic features, highly correlated features were removed using Spearman correlation analysis with a threshold of |r| > 0.9.

Subsequently, feature standardization was performed using StandardScaler, and feature selection was conducted using L1-regularized logistic regression, implemented via the SelectFromModel function in the scikit-learn library, within the cross-validation loop to prevent potential data leakage.

Classical classifiers such as logistic regression (LR), support vector machine (SVM), and random forest (RF) are widely used in medical ML studies and have also been applied in studies involving relatively small and potentially imbalanced datasets [44,45]. DL generally requires large amounts of training data for optimal performance [46]. In this study, to prevent data leakage and ensure patient-level independence, only a single representative CT slice per patient was used, which further limited the dataset size. Therefore, ML approaches were considered more appropriate for this study, and these three models were selected. LR was used as a baseline model due to its interpretability [47], SVM was selected for its robustness and strong generalization ability in high-dimensional feature spaces [48], and RF was adopted as an ensemble method that is robust to noise and effective in handling complex data structures [49].

After feature selection, three ML models, LR, SVM, and RF, were evaluated. All models were trained with class-balanced weights to mitigate the effect of class imbalance.

The radiomic features extracted from CT images converted to Hounsfield units (HU) were used for model training.

No additional intensity normalization was applied to the CT images; however, feature standardization was performed within the ML pipeline using StandardScaler.

Hyperparameters were determined based on empirical evaluation within the cross-validation framework to balance model complexity and avoid overfitting and were kept fixed during all cross-validation folds. The hyperparameters used for each model are summarized in Table 2.

Model performance was evaluated using stratified fivefold cross-validation. The predictive performance of each model was assessed using multiple evaluation metrics, including the AUC, sensitivity, specificity, precision–recall area under the curve (PR-AUC), and F1-score.

To enhance the interpretability of the best-performing model, SHAP was used. SHAP, which is based on Shapley values from cooperative game theory, quantifies the contribution of each input feature to the model prediction [32]. A SHAP summary plot was used to visualize the relative importance of radiomic features contributing to sarcopenia classification.

### 2.6. Statistical Analysis

The performance of each ML model was evaluated separately for APCT and LDCT images using several metrics, including accuracy, sensitivity, specificity, AUC, PR-AUC, and F1-score. Among these metrics, AUC was used as the primary criterion for selecting the optimal model and for overall performance comparison. To estimate the statistical uncertainty of model performance, bootstrap resampling with 2000 iterations was applied to compute 95% confidence intervals for each evaluation metric.

## 3. Results

The diagnostic performance of three ML models—LR, SVM, and RF—was evaluated and compared using the APCT and LDCT datasets. The evaluation metrics included accuracy, sensitivity, specificity, F1-score, PR-AUC, and AUC. AUC and PR-AUC were utilized as the main criteria for selecting the optimal model considering class imbalances in the dataset. The performance of each model is summarized as follows:

For the APCT dataset, the RF model achieved an AUC of 0.720 (95% CI: 0.532–0.881) and a PR-AUC of 0.566 (95% CI: 0.322–0.774). The corresponding accuracy, sensitivity, specificity, and F1-score were 0.802 (95% CI: 0.707–0.880), 0.622 (95% CI: 0.375–0.867), 0.850 (95% CI: 0.754–0.934), and 0.565 (95% CI: 0.345–0.745), respectively.

For the LDCT dataset, the random forest model yielded an AUC of 0.692 (95% CI: 0.573–0.801) and a PR-AUC of 0.940 (95% CI: 0.895–0.975). The corresponding accuracy, sensitivity, specificity, and F1-score were 0.604 (95% CI: 0.518–0.683), 0.570 (95% CI: 0.481–0.655), 0.836 (95% CI: 0.631–1.000), and 0.714 (95% CI: 0.638–0.784), respectively.

A detailed comparison of the performances across all models, including PR-AUC values, is presented in Table 3 and Table 4.

**Figure 4:**
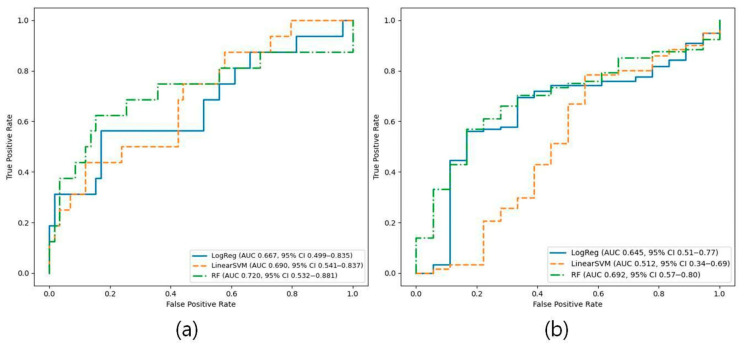
Receiver operating characteristic (ROC) curves of the ML models for sarcopenia classification in (**a**) APCT and (**b**) LDCT datasets.

Figure 4 presents the ROC curves of the ML models for sarcopenia classification using the APCT and LDCT datasets.

**Figure 5:**
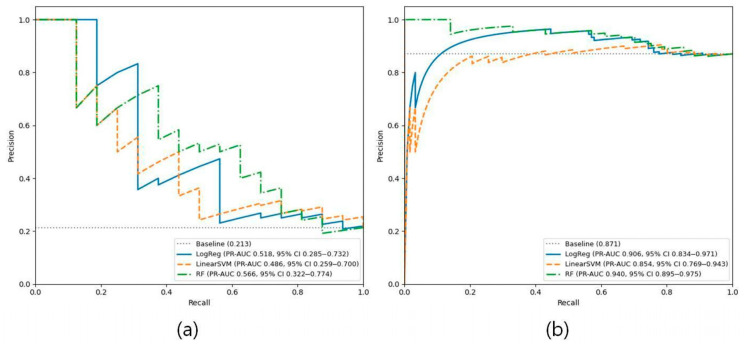
PR curves demonstrating model performance under class imbalance for (**a**) APCT and (**b**) LDCT datasets. The dashed line indicates the no-skill baseline (random precision).

Figure 5 presents the precision–recall (PR) curves of the ML models for sarcopenia classification using the APCT and LDCT datasets.

**Figure 6:**
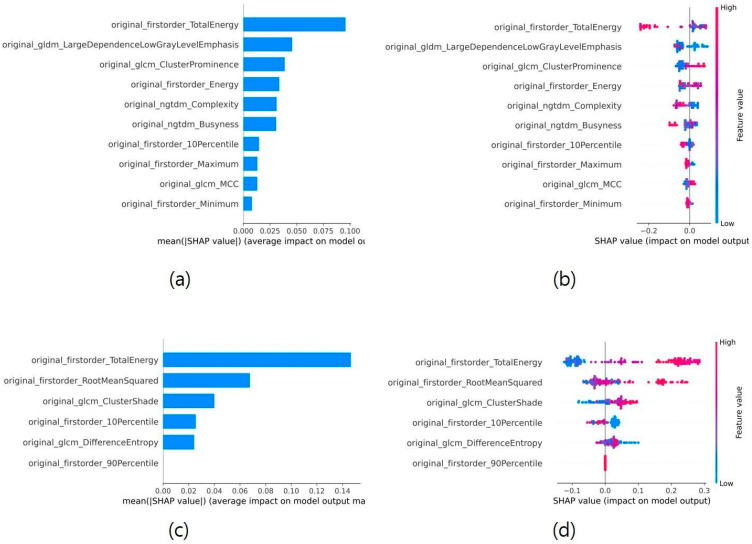
SHAP-based interpretation of the random forest model for sarcopenia classification: (**a**) SHAP feature importance bar plot for the APCT dataset. (**b**) SHAP summary plot for the APCT dataset. (**c**) SHAP feature importance bar plot for the LDCT dataset. (**d**) SHAP summary plot for the LDCT dataset. Features are ranked along the y-axis according to their importance, with the most influential features at the top. Each point represents an individual sample. The x-axis represents the SHAP value, indicating the direction and magnitude of each feature’s contribution to the model output. The color represents the feature value, with red indicating high values and blue indicating low values.

Figure 6 presents SHAP-based visualizations from the random forest model, including feature importance bar plots and SHAP summary plots, illustrating the contribution of radiomic features to sarcopenia classification in the APCT and LDCT datasets.

SHAP analysis provides both global and local interpretability of the model. The feature importance bar plots reflect global interpretability by identifying the most influential radiomic features across the dataset, whereas the SHAP summary plots provide local interpretability by illustrating how individual feature values contribute to model predictions for each sample.

## 4. Discussion

This study aimed to investigate whether radiomic features extracted from LDCT images can be used to identify sarcopenia and to explore the potential applicability of LDCT-based radiomics in clinical assessment. To achieve this objective, radiomic features were extracted from APCT and LDCT images, and ML models were employed to classify sarcopenia. We further employed SHAP-based interpretability analysis to investigate the contributions of individual radiomic features. To focus on radiomic information derived from the original images, image enhancement techniques such as generative adversarial networks (GANs) and denoising algorithms were not applied during preprocessing.

In the APCT dataset, original_firstorder_TotalEnergy was identified as the most important radiomic feature. This feature represents the overall magnitude of voxel intensities within the skeletal muscle region and may reflect global changes in muscle density and tissue composition associated with sarcopenia. The second most important feature, original_gldm_LargeDependenceLowGrayLevelEmphasis, quantifies the presence of large homogeneous regions composed of low-intensity voxels and may correspond to areas of reduced muscle density or fatty infiltration within skeletal muscle. In addition, texture-based features such as original_glcm_ClusterProminence, original_ngtdm_Complexity, and original_ngtdm_Busyness capture asymmetry, local intensity variability, and structural irregularities in gray-level distribution. These features may reflect heterogeneous structural alterations in skeletal muscle during the progression of sarcopenia.

In the LDCT dataset, original_firstorder_TotalEnergy was identified as the most important radiomic feature. This feature reflects the overall magnitude of voxel intensities within the skeletal muscle region and may indicate global changes in muscle density and intensity associated with sarcopenia. This feature was also identified as a key radiomic feature in the APCT dataset, suggesting its relevance to skeletal muscle density assessment across different CT acquisition protocols. The original_firstorder_RootMeanSquared was the second most important feature and represents the average magnitude of voxel intensities, which may reflect overall intensity variations within skeletal muscle tissue. Furthermore, texture-based features such as original_glcm_ClusterShade and original_glcm_DifferenceEntropy describe asymmetry and randomness in gray-level distribution, potentially reflecting structural irregularities of skeletal muscle associated with sarcopenia.

These observations suggest that APCT-based models may capture relatively finer texture heterogeneity within skeletal muscle, whereas LDCT-based models appear to rely more on global intensity-related characteristics.

Interestingly, the same feature, original_firstorder_TotalEnergy, emerged as the most influential predictor in both the APCT- and LDCT-based models.

Although the two datasets were analyzed independently, this observation suggests that radiomic characteristics related to overall muscle intensity may represent a potentially shared imaging pattern associated with sarcopenia across different CT acquisition conditions. This finding further supports the potential feasibility of LDCT-based radiomics for sarcopenia assessment despite differences in imaging conditions.

In this study, radiomic features extracted from APCT and LDCT images were used to train ML models for sarcopenia classification. Performance was evaluated using multiple metrics, including accuracy, sensitivity, specificity, F1-score, PR-AUC, and AUC. We acknowledge that the relatively small sample size and class imbalance could potentially lead to biased performance estimates and wide confidence intervals, reflecting statistical uncertainty. To mitigate these effects and ensure a robust evaluation, we utilized evaluation metrics that are more informative under class imbalance, such as PR-AUC and F1-score. In particular, PR-AUC focuses on the performance of the positive class and is sensitive to class prevalence, making it more appropriate than AUC in imbalanced settings.

For the APCT dataset, the random forest (RF) model demonstrated the best overall performance, achieving an AUC of 0.720 (95% CI: 0.532–0.881) and a PR-AUC of 0.566 (95% CI: 0.322–0.774). The corresponding accuracy, sensitivity, specificity, and F1-score were 0.802 (95% CI: 0.707–0.880), 0.622 (95% CI: 0.375–0.867), 0.850 (95% CI: 0.754–0.934), and 0.565 (95% CI: 0.345–0.745), respectively.

For the LDCT dataset, the RF model also showed the best performance, with an AUC of 0.692 (95% CI: 0.573–0.801) and a PR-AUC of 0.940 (95% CI: 0.895–0.975). The corresponding accuracy, sensitivity, specificity, and F1-score were 0.604 (95% CI: 0.518–0.683), 0.570 (95% CI: 0.481–0.655), 0.836 (95% CI: 0.631–1.000), and 0.714 (95% CI: 0.638–0.784), respectively. The relatively high PR-AUC observed in the LDCT dataset may be influenced by the underlying class distribution and should be interpreted with caution.

These findings suggest that LDCT-derived radiomic features may contain information relevant to sarcopenia assessment, even under conditions of reduced radiation dose and increased image noise.

Previous studies have investigated CT-based radiomics for sarcopenia assessment using ML and DL approaches [9,14,17]. In particular, Vogele et al. [9] applied CT radiomics combined with ML for sarcopenia classification and further evaluated its association with disease progression in cancer patients.

In contrast, the present study aimed to assess whether radiomic features extracted from LDCT images can preserve sufficient information for sarcopenia classification.

Although LDCT images show more noise and lower visual quality than standard CT, radiomics analysis extracts statistical and textural tissue patterns that may support ML models. As slice thickness changes, image noise and diagnostic image quality may be affected [50]. Sanchez et al. reported that radiomic features remained relatively stable across various slice-thickness conditions, particularly for muscle tissues, demonstrating high reproducibility [51]. Zwanenburg et al. also reported that many radiomic features showed high reproducibility across different imaging conditions, suggesting that radiomics may provide robust quantitative information even in the presence of variations in image quality [52]. These findings suggest that radiomics may provide quantitative information even under noisy or degraded image quality conditions.

Moreover, LDCT images are characterized by increased noise and decreased SNR due to reduced radiation exposure [53], which, similar to reduced slice thickness, can negatively affect image quality. Although the sources differ, both factors potentially lower image fidelity.

Empirical evidence supports the feasibility of radiomics-based muscle analysis and diagnostic predictions using LDCT. Lim et al. reported a high correlation (R^2^ = 0.74–0.79) between muscle area measurements—such as L1-SMA and pectoralis muscle—from chest LDCT and L3-SMA from standard-dose CT, confirming the reliability of muscle quantification using LDCT [54]. Liu et al. conducted a comparative study using LDCT and standard-dose CT (SDCT) images acquired under the same conditions, extracting radiomic features, and building ML models. Their LDCT-based model achieved an AUC of 0.915 (validation AUC, 0.976) and showed no statistically significant difference from the SDCT model, with higher specificity than the Lung-RADS criteria [29].

These results suggest that LDCT images may provide radiomic features informative for ML-based sarcopenia classification. Our study evaluated the feasibility of using LDCT, a low-radiation alternative to APCT, for sarcopenia assessment and demonstrated the potential applicability of LDCT-derived radiomic features in this context.

However, several limitations should be acknowledged.

First, the dataset used in this study was relatively small and exhibited class imbalance between sarcopenia and non-sarcopenia cases. Although class weighting was applied during model training to mitigate this issue, and the model’s predictive viability was further assessed through PR-AUC analysis to account for the skewed distribution, further studies using larger datasets with balanced class distributions are required to improve model robustness.

Second, model performance was evaluated using internal cross-validation on data collected from a single institution, without external validation using independent datasets. As imaging protocols and patient characteristics may vary across institutions, future studies incorporating multi-center datasets and external validation are needed to further assess the generalizability of the proposed approach.

Third, the APCT and LDCT images were not obtained from the same individuals; therefore, the models for each modality were developed independently. In addition, different anatomical reference levels were used for sarcopenia assessment, with L3 used in APCT and L1 used in LDCT, which may introduce variability in radiomic feature distributions and affect direct comparability between modalities. A more direct comparison of radiomics-based sarcopenia assessment between these modalities would require paired APCT and LDCT datasets from the same patients. Such an approach would allow for a more controlled evaluation of intermodality differences and diagnostic consistency.

Fourth, although ROI annotation was performed by experienced radiologists with consensus resolution, inter-observer reliability metrics such as the intraclass correlation coefficient (ICC) or Dice similarity coefficient were not formally assessed. The absence of these quantitative measures for reproducibility is a limitation of the current study.

Future studies with larger, multi-center, and paired datasets are warranted to further evaluate model robustness and clinical applicability.

## 5. Conclusions

This study investigated whether radiomic features extracted from LDCT images retain meaningful information associated with sarcopenia. Radiomic features extracted separately from APCT and LDCT images were used to construct classification models, and SHAP-based analysis was performed to evaluate the contribution of key features.

The results suggest that radiomic features extracted from LDCT images may contain informative patterns associated with sarcopenia. Despite the lower radiation dose and increased image noise associated with LDCT acquisition, radiomics-based ML models showed imaging patterns associated with skeletal muscle characteristics.

These findings indicate the potential applicability of LDCT-derived radiomic features for sarcopenia assessment. Considering that LDCT is already widely used in clinical practice, particularly for lung cancer screening, LDCT-based radiomics may contribute to opportunistic sarcopenia assessment as an exploratory approach. Importantly, because LDCT involves lower radiation exposure than standard CT, it may provide a potentially useful option for elderly patients and individuals with chronic conditions who require repeated imaging examinations.

## Figures and Tables

**Table 1:** Baseline clinical characteristics of the study cohort stratified by imaging modality and sarcopenia status.

Cohort	Group	N	Age (Years)	Male, n (%)	Female, n (%)
APCT	Sarcopenia	16	57.75 ± 9.79	11 (68.8%)	5 (31.2%)
	Normal	59	54.73 ± 8.26	35 (59.3%)	24 (40.7%)
LDCT	Sarcopenia	121	56.60 ± 8.81	90 (74.4%)	31 (25.6%)
	Normal	18	52.83 ± 6.66	1 (5.6%)	17 (94.4%)

**Table 2:** Model configuration and fixed hyperparameters used in this study.

Component	Hyperparameter	Value
Feature Selector (L1-regularized logistic regression via SelectFromModel)	Regularization (C)	0.9
LR	Class weight	balanced
	Solver	liblinear
	Penalty	L2
SVM	Calibration method	Sigmoid (Platt scaling)
	Class weight	balanced
RF	Number of estimators	600
	Max depth	3
	Min samples leaf	5
	Class weight	balanced

**Table 3:** Performance metrics of ML models using APCT images.

Model	AUC (95% CI)	PR-AUC (95% CI)	Accuracy (95% CI)	Sensitivity (95% CI)	Specificity (95% CI)	F1-Score (95% CI)
LR	0.667 (0.499–0.835)	0.518 (0.285–0.732)	0.772 (0.680–0.867)	0.561 (0.308–0.812)	0.829 (0.732–0.917)	0.505 (0.276–0.698)
SVM	0.690 (0.541–0.837)	0.486 (0.259–0.700)	0.786 (0.693–0.880)	0.436 (0.188–0.700)	0.882 (0.793–0.964)	0.457 (0.222–0.667)
RF	0.720 (0.532–0.881)	0.566 (0.322–0.774)	0.802 (0.707–0.880)	0.622 (0.375–0.867)	0.850 (0.754–0.934)	0.565 (0.345–0.745)

**Table 4:** Performance metrics of ML models using LDCT images.

Model	AUC (95% CI)	PR-AUC (95% CI)	Accuracy (95% CI)	Sensitivity (95% CI)	Specificity (95% CI)	F1-Score (95% CI)
LR	0.645 (0.506–0.772)	0.906 (0.834–0.971)	0.597 (0.511–0.676)	0.562 (0.472–0.650)	0.831 (0.625–1.000)	0.707 (0.630–0.776)
SVM	0.512 (0.342–0.686)	0.854 (0.769–0.943)	0.740 (0.662–0.813)	0.785 (0.708–0.855)	0.444 (0.200–0.684)	0.839 (0.783–0.889)
RF	0.692 (0.573–0.801)	0.940 (0.895–0.975)	0.604 (0.518–0.683)	0.570 (0.481–0.655)	0.836 (0.631–1.000)	0.714 (0.638–0.784)

## Data Availability

The datasets generated and analyzed during the current study are not publicly available due to institutional and ethical restrictions.
